# Quantitative susceptibility mapping demonstrates ageing-related long-term structural brain changes in adult rats after mild ischaemic stroke

**DOI:** 10.1093/braincomms/fcag182

**Published:** 2026-05-21

**Authors:** Zuitian Tao, Charlotte M Ermine, David K Wright, Mohamed Salah Khlif, Vanessa H Brait, Lachlan H Thompson, Leigh A Johnston, Amy Brodtmann, Warda T Syeda

**Affiliations:** Department of Biomedical Engineering, The University of Melbourne, Parkville, Victoria 3010, Australia; The Florey Institute of Neuroscience and Mental Health, Parkville, Victoria 3010, Australia; The Department of Neuroscience, School of Translational Medicine, Monash University, Melbourne, Victoria 3004, Australia; Cognitive Health Initiative, School of Translational Medicine, Monash University, Melbourne, Victoria 3004, Australia; The Florey Institute of Neuroscience and Mental Health, Parkville, Victoria 3010, Australia; The Florey Institute of Neuroscience and Mental Health, Parkville, Victoria 3010, Australia; Department of Biomedical Engineering, The University of Melbourne, Parkville, Victoria 3010, Australia; Melbourne Brain Centre Imaging Unit, The University of Melbourne, Parkville 3010, Victoria, Australia; Cognitive Health Initiative, School of Translational Medicine, Monash University, Melbourne, Victoria 3004, Australia; Melbourne Brain Centre Imaging Unit, The University of Melbourne, Parkville 3010, Victoria, Australia; Department of Radiology, The University of Melbourne, Parkville 3010, Victoria, Australia

**Keywords:** stroke, quantitative susceptibility mapping, rat models, MRI, brain

## Abstract

Quantitative susceptibility mapping is a novel, non-invasive MRI technique that measures tissue magnetic susceptibility, offering insights into brain iron, calcium and myelin distribution. Here, we used quantitative susceptibility mapping to assess whole-brain long-term susceptibility changes in adult rats following endothelin-1-induced mild focal stroke in the right motor cortex over 48 weeks. No significant differences in susceptibility trajectories were found between stroke and sham groups in either hemisphere. However, both groups exhibited shared regional susceptibility changes, likely age-related, reflecting variations in iron, calcium, or white matter myelination over time. These findings provide a new perspective on ageing effects on brain metal accumulation and structural integrity, highlighting the need for further histological validation to clarify underlying mechanisms.

## Teaser

Tao *et al*. report that longitudinal magnetic resonance imaging in an experimental stroke model revealed distinct brain regions showing both increases and decreases in tissue susceptibility over time. Their findings highlight region-specific changes linked to ageing and stroke, providing insight into mechanisms of brain injury and recovery.

## Graphical Abstract

**Graphical Abstract:**
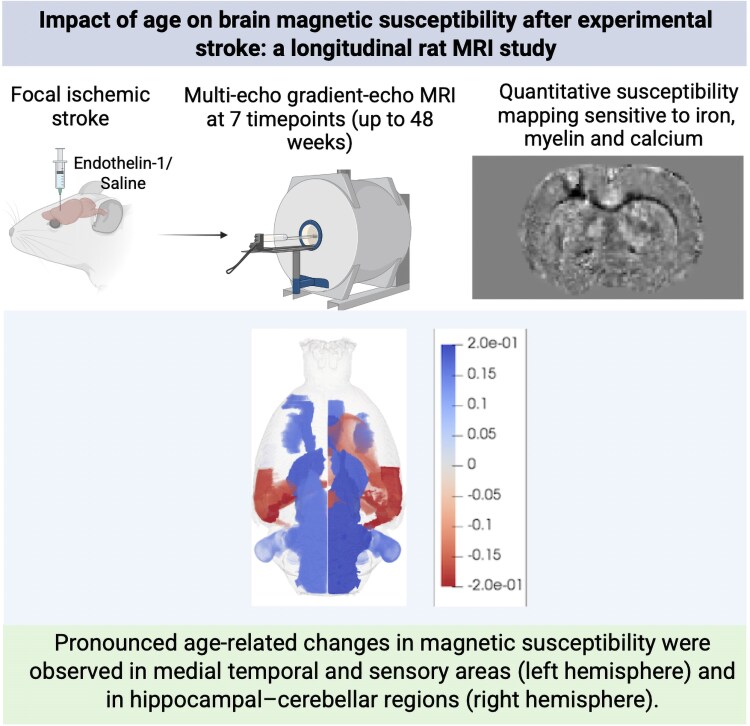

## Introduction

Stroke is a leading cause of disability and death globally, often leading to secondary neurodegeneration in brain regions connected to the primary injury site.^1-4^ Studies have reported secondary degeneration in the cortex,^1,5-7^ hippocampus,^8-11^ thalamus,^12-16^ and corpus callosum,^17^ which may contribute to post-stroke functional and cognitive impairments, accelerated atrophy and vascular dementia.^6,7,11,16,18-20^ Even minor ischaemic events, such as transient ischaemic attacks (TIAs), can cause long-term brain changes, including atrophy^21^ and microvascular damage,^22,23^ leading to stroke, TIA and cognitive decline risks.^24-26^ However, the pathological mechanisms underlying brain changes after a minor stroke or TIA remain poorly understood.

Animal studies are important for understanding secondary injuries after ischaemic stroke. The middle cerebral artery occlusion (MCAO) model is the most used model for studying stroke. However, MCAO can cause large infarcts involving both subcortical and cortical structures,^27^ and can preclude the precise localization of secondary degeneration.^27^ In contrast, the endothelin-1 (ET-1) model uses intracranial ET-1 delivery to induce precisely localized strokes, making it ideal for studying remote neurodegeneration mechanisms following minor strokes.^28-31^

Iron and calcium are vital bio-metals in the brain, essential for neuronal function. Studies link iron accumulation to ischaemic injury, with iron deposits observed in the cortex and hippocampus post-TIA in animals and ischaemic regions in human.^32-37^ High plasma ferritin correlates with poor stroke outcomes, highlighting disrupted iron homeostasis and its role in neuronal injury.^38,39^ Ischaemic stroke also affects calcium balance, triggering neuronal death via excitatory neurotransmitter release.^40-42^ Additionally, ischaemic stroke damages white matter, causing demyelination that contributes to long-term motor and cognitive impairments.^43,44^ However, the long-term impacts of iron, calcium and demyelination changes after stroke remain unclear.

Paramagnetic iron, diamagnetic myelin and calcium are key sources of magnetic susceptibility in the brain.^45,46^ Quantitative susceptibility mapping (QSM) is an MRI technique that measures tissue magnetic susceptibility, enabling non-invasive monitoring of iron, calcium and myelin changes. QSM-based iron estimates have been validated through post-mortem techniques,^47-49^ and studies show QSM can differentiate calcifications from iron in microbleeds.^50-54^ Magnetic susceptibility also serves as a biomarker for myelin, with QSM detecting susceptibility shifts correlated with myelin levels in both human and animal studies.^55-58^

Mapping changes in brain iron, calcium and myelin can clarify the long-term effects of minor stroke. However, longitudinal imaging studies yield high-dimensional data, even with small samples. Data dimensionality is determined by the number of features (voxels or regions-of-interest, ROIs) and time points. These datasets often contain spurious fluctuations that require filtering before statistical analysis. Dimensionality reduction techniques, like principal component analysis (PCA), are commonly used to remove noise while preserving biologically relevant signals.^59-62^ However, longitudinal variants of PCA are based on two-dimensional matrix-based algebra and do not treat time as a separate dimension.^63,64^ Tensor-based methods can model time as a separate dimension, making them effective for analysing high-dimensional, multiway data in longitudinal studies, such as microbiome^63,65-67^ and neural dynamics.^68^ The tensor-based tensor component analysis (TCAM) technique, in particular, overcomes PCA limitations, using tensor algebra to identify key biological signals.^63,69^

In the current study, we used QSM to investigate long-term magnetic susceptibility changes in adult rats after ET-1-induced mild focal stroke in the right motor cortex over a clinically relevant period of 48 weeks. This injection site was selected because focal lesions in the motor cortex are well established in the literature, produce reproducible motor deficits and allow examination of secondary neurodegeneration in connected cortical and subcortical networks, while avoiding the large, variable infarcts seen with MCAO models. Patterns of regional susceptibility changes over time were assessed using TCAM which identifies lower-dimensional spatiotemporal components in the data, such that each component is a weighted combination of the original variables measured across time. We have previously reported long-term cortical and subcortical volume changes and white matter atrophy using structural and diffusion MRI in this cohort.^20^ Here, we hypothesized the emergence of long-term magnetic susceptibility changes in brain areas connected to the infarcted motor cortex, due to iron and calcium accumulation and white matter changes.

## Materials and methods

### Animals

The Animal Ethics Committee of The Florey Institute approved the study. Adult 22-week-old male Long Evans rats were housed under a 12-h light/dark cycle with ad libitum access to food and water, following the Stroke Therapy Academic Industry Roundtable guidelines.^70^

### Experimental focal ischaemic stroke

Focal ischaemia was induced by ET-1 injection to the motor cortex of rats at 22 weeks of age. Prior to surgery, animals were anaesthetized with isoflurane (5% at 1 L/min) and placed in a stereotaxic frame (Kopf, Germany) where deep anaesthesia was maintained for the duration of the surgery (2% at 1 L/min). The ET-1 toxin (800 pmol, Auspep) for the lesioned animals (*n* = 14) or saline for the controls (*n* = 10) was injected into the motor cortex at two rostro-caudal locations: 0.5 and 2.0 mm rostral and 2.8 mm lateral to bregma and 1.5 mm below the surface of the brain. One control rat was sacrificed during the study due to the presence of a tumour growth and was excluded from the analysis. All rats were sacrificed 48 weeks post-procedure in the last *in vivo* MRI experiments.

### MRI experiments

For MRI, rats were anaesthetised with isoflurane and placed in a rat cradle with tooth and ear-bars to fix head position. During scanning, rats were kept anesthetized with a mixture of 1–2% isoflurane and oxygen. A small air balloon attached to a pressure transducer was placed under the chest to monitor respiration. Body temperature was continuously observed using a rectal probe and kept at 37°C via a hot water circulation system. MRI was performed *in vivo* at baseline (0 week), 1, 4, 12, 24, 36 and 48 weeks post-stroke. At the 24-week time point, scans for three animals (1 sham, 2 ET-1) were incomplete due to technical issues; these animals were therefore excluded from the study.

MRI was performed using a 4.7T MRI with Avance III console and rat surface coil (Bruker, USA). Multi-echo T_2_*-weighted images were acquired using a 3D sequence with parameters: first echo time = 4 ms, echo-spacing = 4 ms, 20 echoes, repetition time = 110 ms, matrix size = 176 × 128 × 70 and 0.15 mm isotropic resolution. Raw multichannel k-space data were exported from the scanner and magnitude and phase images were reconstructed in MATLAB.

### Quantitative susceptibility mapping

**Figure 1:**
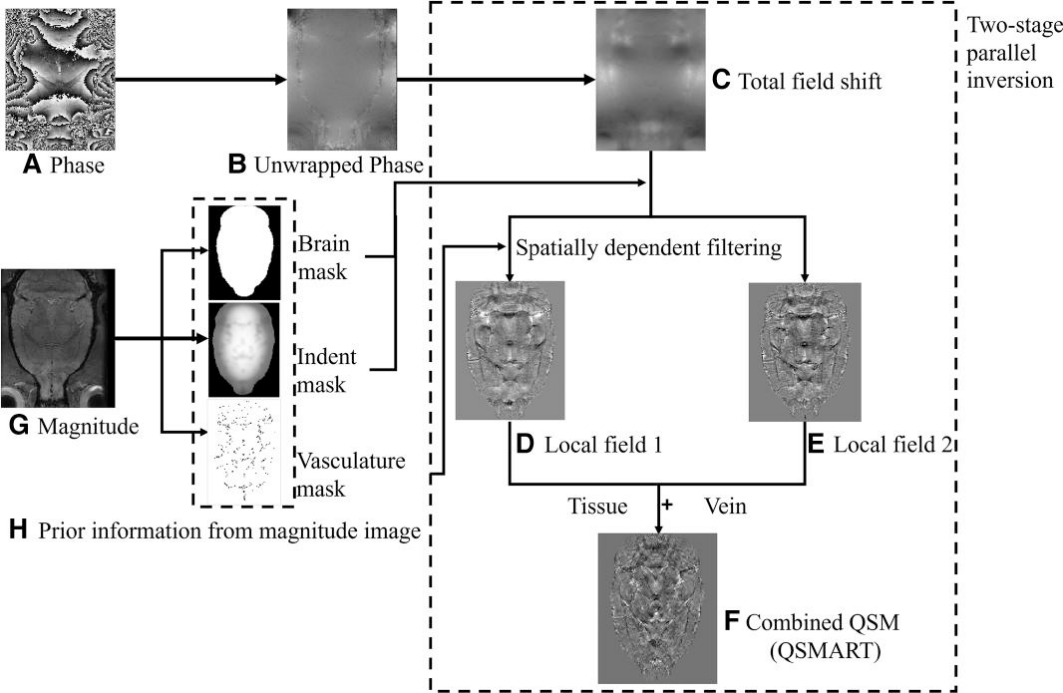
**QSMART reconstruction pipeline.** (**A**) Raw phase image (after coil combination). (**B**) Unwrapped phase. (**C**) Total field shift map. Prior information derived from the magnitude image is used to guide subsequent processing: (**G**) magnitude image and (**H**) corresponding masks (brain mask, indent mask and vasculature mask). The total field shift map undergoes spatially dependent filtering to generate two local field components, (**D**) local field 1 (tissue) and (**E**) local field 2 (vein), which are then input to a two-stage parallel inversion to produce (**F**) the combined susceptibility map.

All magnitude multi-echo-T2*w images were bias-field corrected and averaged across echoes to increase contrast. The QSM map for each subject was created from the multi-echo magnitude and phase images using the quantitative susceptibility mapping artefact reduction technique (QSMART).^71^ Briefly, the QSMART pipeline includes coil combination, phase unwrapping, spatially dependent filtering and two-stage parallel inversion to extract susceptibility maps from MRI phase data (Fig. 1).

QSMART parameters were adjusted for the rat data: Frangi filter scale range = 0.01–0.4, Frangi filter step-size = 0.1, Frangi filter sensitivity thresholds *α, β, c* = 0.5, 0.5, 60, spatial dependent filtering *σ__C_*, *σ__V_* = 10 and 2, two curvature-specific parameters: lower limit = 0.6, curvature constant = 100. To account for the arbitrary offset in QSM reconstructions, susceptibility values were referenced to the mean of the contralesional hemisphere at each time point, selected as an internal reference region for consistency across animals and time points.

### ROI segmentation

An atlas-based approach was used to segment ROIs for each subject using the ANTs software package.^72^ For each cohort and time-point, an intermediate template was separately constructed from the T_2_*-weighted images, and these intermediate templates were used to construct an unbiased study template.^73^ The study template was registered nonlinearly to the SIGMA rat brain atlas,^74^ which contains 98 ROIs in total (encompassing both grey matter and major white matter structures). A full list of ROIs from the atlas can be found in the Supplementary material. Inverse transforms were used to segment these ROIs in the study template space. Combined inverse diffeomorphisms from study template to each individual space were used to segment ROIs in the individual space. Region volume for each subject in each ROI was computed by voxel-count, and the average susceptibility for each subject in each ROI was computed.

### Statistical analyses

All statistical analyses were performed using the individual animal as the experimental unit. Regional mean magnetic susceptibility and volume values were averaged within each ROI per animal at each time point before group-level analyses.

*Z*-score normalization was performed on regional volume and magnetic susceptibility data in each ROI relative to the mean and standard deviation of the entire cohort (stroke and sham combined) to minimize inter-ROI variability and facilitate comparisons across all subjects. TCAM-based dimensionality reduction was performed on the normalized longitudinal data, with missing values imputed by the TCAM framework. Similar to a PCA analysis, TCAM outputs consist of factors and loadings (or importance scores). TCAM factors are latent variables that represent the 98-dimensional (98 ROIs) QSM trajectories of subjects. TCAM loadings represent the degree to which each ROI contributes to the latent factor, and are derived across the entire cohort (stroke and control animals), thereby reflecting common covariance patterns in the data.

The first two TCAM factors explaining the most variance in the data were used to construct a two-dimensional latent space, where temporal trajectories from all regions for each subject were represented as a single point. This approach is consistent with prior dimensionality reduction studies, where higher-order components often reflect noise or individual variation rather than robust biological signal.^61,75^

A region-wise linear mixed effects model was implemented to examine region-wise volume and magnetic susceptibility changes over time. Regions were selected using a TCAM-based pruning strategy: regions with TCAM loading exceeding an arbitrary threshold of 0.12 were included in analyses. Time was included as a continuous independent variable in the model and mean regional magnetic susceptibility as the dependent variable. The regional magnetic susceptibility at baseline was included as a covariate. To address between-subject heterogeneity, correlated random intercept and random slope (time point) grouped by subject were included in the model as random effects. A *P*-value < 0.05 was considered statistically significant and was corrected for multiple comparisons using Benjamini and Hochberg FDR correction.^76^

## Results

### Long-term susceptibility changes

**Figure 2:**
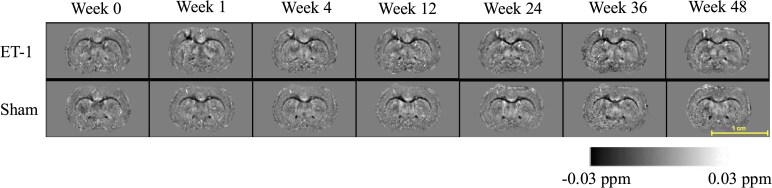
**ET-1 induced stroke progression from baseline to 48 weeks post-surgery.** QSM images (values shown in parts per million, ppm) show the injection site (top row) at Week 1 and capture lesion evolution over time.

**Figure 3:**
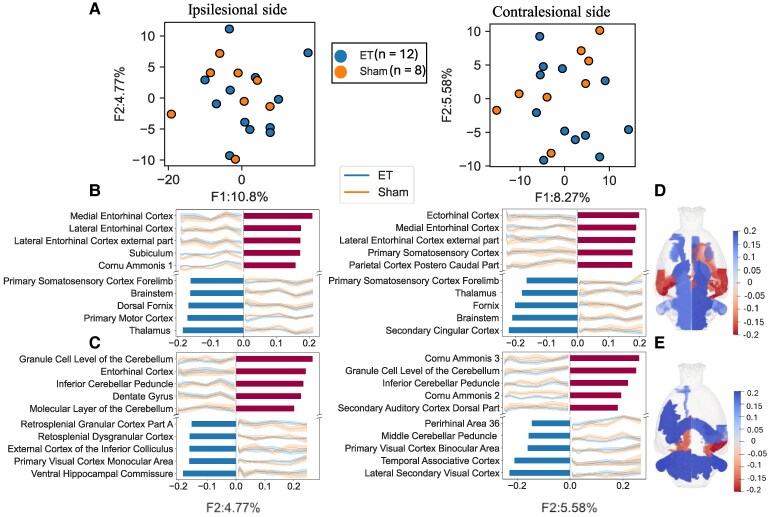
**Top contributing ROIs in leading TCAM factors,** F1 and F2, and their loadings (importance score). **(A)** TCAM factors in the reduced two-dimensional QSM space in the ipsilesional hemisphere and contralesional hemisphere. Each point represents one animal’s latent TCAM factor scores (F1, F2) derived from TCAM applied to the full longitudinal dataset (all time points compressed into a single score per animal, capturing the evolution of regional QSM across time). Two-sample independent *t*-tests on F1 and F2 for each hemisphere showed no significant differences between endothelin-1 (ET-1) and sham groups (ipsi-F1: *t* = 1.33, *P* = 0.198, ipsi-F2: *t* = −0.30, *P* = 0.763, contra-F1: *t* = 0.67, *P* = 0.512, contra-F2: *t* = −1.25, *P* = 0.226). **(B)** Bar graph shows leading ROIs contributing to F1 of QSM in the ipsilesional (left) and contralesional (right) hemispheres from 12 ET-1 and 9 sham rats, with strong contributions from the medial entorhinal cortex, lateral entorhinal cortex, thalamus and primary somatosensory cortex. **(C)** Bar graph shows leading ROIs contributing to F2 of QSM in the ipsilesional (left) and contralesional (right) hemispheres from 12 ET-1 and 9 sham rats, with strong contributions from the granule cell level of the cerebellum, inferior cerebellar peduncle, cornu Ammonis 3 and middle cerebellar peduncle. The trajectories represent the mean QSM from baseline (0 week) to 48 weeks in sham rats (orange) and stroke rats (blue). The shaded regions of the trajectories represent the standard error of the mean **(D)** Glass brain showing top contributing ROIs in F1, coloured by corresponding TCAM loadings. **(E)** Glass brain showing top contributing ROIs in F2, coloured by corresponding TCAM loadings.

The ET-1 injection site in the right motor cortex was visible on QSM images. Figure 2 shows exemplar QSM images of a stroke rat and a sham rat in study template space, displayed in radiological convention. To investigate the temporal trajectories of regional QSM, TCAM was implemented separately in ipsilesional and contralesional hemispheres to construct a reduced two-dimensional space in each hemisphere. The two leading factors (see Fig. 3A), F1 and F2, explained most variance in the ipsilesional hemisphere (10.6% and 4.8%) and in the contralesional hemisphere (8% and 5.62%). As shown in Fig. 3A, each point represents a QSM temporal trajectory of a subject in the reduced space. Inspection of the top contributing ROIs indicated that F1 primarily represented medial temporal and primary sensory cortical regions, whereas F2 was driven by hippocampal and cerebellar contributions.

A *t*-test performed on the TCAM factors showed no significant difference between the ET and Sham groups on ipsilesional (F1: t = 1.33, *P* = 0.198, F2: *t* = −0.30, *P* = 0.763) and contralesional sides (F1: *t* = 0.67, *P* = 0.512, F2: *t* = −1.25, *P* = 0.226), suggesting no significant between-group effects of mild ischaemic stroke on the QSM temporal trajectories.

### Susceptibility changes shared between groups suggest age-related iron, calcium and myelin alterations

To further determine the source of the time effect in leading TCAM factors, we considered the top contributing ROIs to the variation in F1 and F2 in the ipsilesional and contralesional hemispheres according to their TCAM loadings, which represent the direction and magnitude of the contribution of each ROI to each of the TCAM factors. The top contributing ROIs and corresponding loading in F1 (Fig. 3D) and F2 (Fig. 3E) are displayed in glass brains. Figure 3B shows the contributing ROIs with top five TCAM loadings to F1 in each hemisphere. Figure 3C shows the contributing ROIs with top TCAM loadings to the F2 in each hemisphere. These regions represent the strongest sources of spatiotemporal variations in the QSM signal.

### Follow-up analyses

A region-wise linear mixed effects model was performed to test QSM changes over time on each ROI with top loadings to F1 and F2 in each hemisphere (Table 1). In the ipsilesional hemisphere, susceptibility significantly increased over time in cornu ammonis 3, while decreased susceptibility was observed in the ectorhinal cortex, interpeduncular nucleus and dorsal fornix after correcting for multiple comparisons. In the contralesional hemisphere, susceptibility significantly increased over time in the perirhinal area 36, entorhinal cortex, granule cell layer of the cerebellum, parietal cortex posterocaudal part, parietal cortex posterorostral part and the primary somatosensory cortex dysgranular zone. In contrast, decreases over time were observed in the anterior commissure posterior part, dorsal fornix, glomerular layer of the accessory olfactory bulb and the interpeduncular nucleus.

**Table 1:** Regions with significant time effects after correcting for multiple comparisons

Region	Hemisphere	*t*-statistic	*P*-value (after multiple comparison correction)
Cornu ammonis 3	Ipsi	6.33	<0.000001
Ectorhinal cortex	Ipsi	−4.00	<0.01
Interpeduncular nucleus	Ipsi	−3.89	<0.01
Dorsal fornix	Ipsi	−2.81	<0.05
Perirhinal area 36	Contra	6.30	<0.0000001
Entorhinal cortex	Contra	4.99	<0.0001
Granule cell level of the cerebellum	Contra	4.80	<0.0001
Anterior commissure posterior part	Contra	−4.50	<0.0001
Dorsal fornix	Contra	−3.77	<0.01
Glomerular layer of the accessory olfactory bulb	Contra	−3.63	<0.01
Interpeduncular nucleus	Contra	−3.46	<0.01
Parietal cortex posterorostral part	Contra	3.19	<0.01
Parietal cortex posterocaudal part	Contra	2.97	<0.05
Primary somatosensory cortex dysgranular	Contra	2.87	<0.05

### Latent dimensions of spatiotemporal volume changes across time

**Figure 4:**
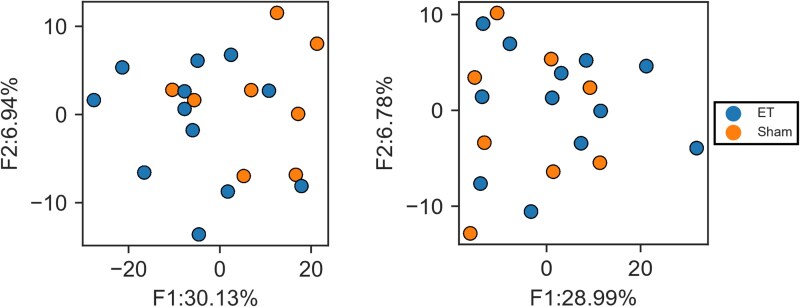
**Distribution of TCAM scores across groups.** Scatter plot of TCAM scores in the reduced two-dimensional volume space of two top TCAM factors in the ipsilesional hemisphere (left) and contralesional hemisphere (right). Each point represents one animal’s latent TCAM factor scores (F1, F2) derived from TCAM applied to the full longitudinal dataset (all time points compressed into a single score per animal, capturing the evolution of regional QSM across time). Two-sample *t*-test found significant group difference in ipsilesional F1 (*t* = −1.37, *P* = 0.029) and no significant differences in ipsilesional F2 (*t* = −0.90, *P* = 0.378), and contralesional factors (F1: *t* = 1.11, *P* = 0.280, F2: *t* = −0.47, *P* = 0.644) between ‘endothelin-1 (ET-1)’ and sham groups.

To investigate the temporal trajectories of rat brain volume, TCAM analysis was performed to construct a reduced multi-dimensional space in each hemisphere. The two leading factors, F1 and F2, explained most variance in the ipsilesional hemisphere (30.13% and 6.94%) and in the contralesional hemisphere (28.99% and 6.78%). A student *t*-test was performed on the TCAM scores and showed a significant difference between the ET and Sham groups in ipsilesional F1 (*t* = −1.37, *P* = 0.029) (Fig. 4) and no difference in ipsilesional F2 (*t* = −0.90, *P* = 0.378). No significant differences were found in F1 and F2 on the contralesional side (F1: *t* = 1.11, *P* = 0.280, F2: *t* = −0.47, *P* = 0.644).

### Latent pattern of volume

**Figure 5:**
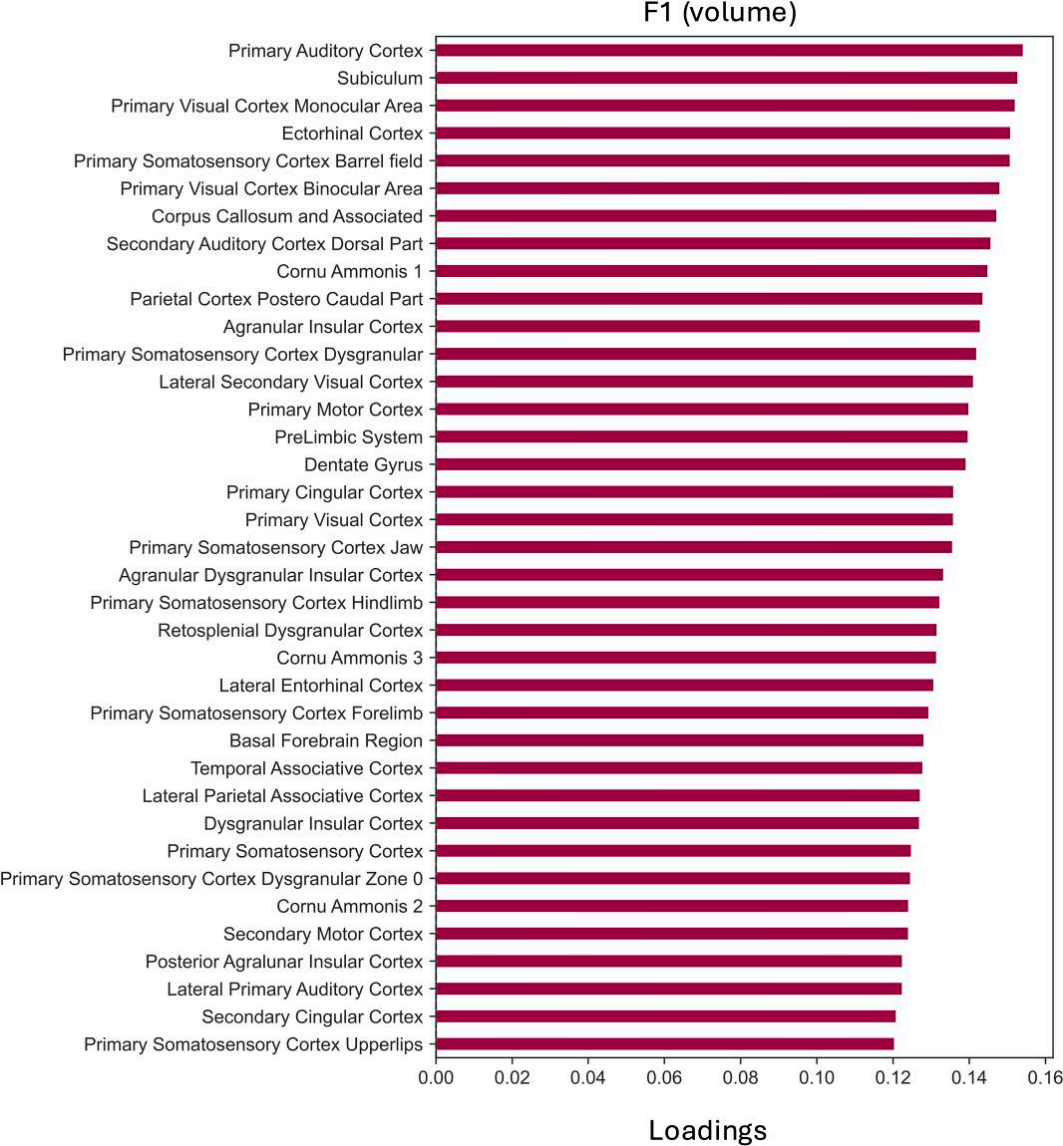
**Glass brain representation of TCAM loadings in factor 1 (F1).** Regions with the highest TCAM loadings in Factor 1 (F1; *x*-axis) contribute to brain volume differences between sham and endothelin-1 (ET-1) groups. The observed regional pattern suggests long-term atrophy in ET-1 rats.

To find the ROIs that contribute to the variations of brain volume between the sham and ET-1 groups, we inspected ROIs with top TCAM loadings in the F1 factor (Fig. 5). Larger brain volumes of ROIs correlated with increased F1 TCAM score, suggesting the potential long-term atrophy of the ROIs in ET-1 rats.

## Discussion

This is the first study to explore long-term QSM changes in rat stroke. We identified multivariate patterns of regional volume and QSM changes over time using a tensor-based dimensionality reduction technique called TCAM. The first regional volume pattern had the strongest contributions from the cortical regions and showed significant differences between the stroke and sham groups; which is consistent with the long-term cortical atrophy we previously reported using univariate analyses.^20,31^ However, TCAM found no significant difference in QSM trajectories between stroke and sham rats in each hemisphere. The results suggest the minor stroke induced by ET-1 does not lead to observable long-term magnetic susceptibility changes. We observed regional QSM changes shared between both sham and stroke groups. These shared QSM changes suggest age-related iron, calcium, or white matter myelination changes over time. Interestingly, ROIs showing significant age-related changes differed between hemispheres. This asymmetry may reflect regional differences in iron accumulation and calcium dyshomeostasis, as well as the selective vulnerability of white matter pathways to ageing processes. The ipsilesional hemisphere may undergo subtle compensatory reorganization after stroke, whereas contralesional regions may follow a more typical ageing trajectory, leading to divergent regional patterns over time.

Variable timing of iron accumulation in rats after stroke has been observed in previous studies. Iron accumulation observed in the rat brain after transient ischaemic stroke in the cortex,^32,33,36^ hippocampus,^32,33,36^ corpus callosum,^33^ and thalamus^77^ by Perl’s staining range from one week to 24 weeks. Myelin loss was also found in the external capsule and striatum of stroke rats by Luxol Fast Blue myelin staining at 4 weeks after stroke.^78^ However, we could not observe these changes in the QSM signal. Several factors could account for this discrepancy. First, the precise location and extent of ET-1–induced stroke lesions may differ between studies, potentially influencing downstream iron and myelin changes. Second, our longitudinal design tracks within-subject changes over 48 weeks, whereas many earlier studies were cross-sectional, which may be more sensitive to transient post-stroke effects. Finally, QSM may be less sensitive than histological methods for detecting microstructural changes in iron or myelin, particularly in small animal models, which could explain the absence of group-level stroke-related differences in our data.

In addition to iron, diamagnetic myelin in the white matter and calcium also contribute to the observed QSM signal. Our previous study in the same cohort found significantly reduced fractional anisotropy in the motor cortex and cingulum bundle regions of stroke rats using diffusion tensor imaging (DTI).^20^ The fractional anisotropy in DTI is sensitive to both geometrical architecture of white matter and myelin.^79,80^ The result of this study shows the stroke does not change the QSM signal, indicating there are no QSM-observable myelin changes. Therefore, the reduced fractional anisotropy found in stroke rats in our previous result is potentially associated more strongly with the geometrical reorganization of white matter than demyelination.

Understanding of iron accumulation and white matter changes during normal ageing remains incomplete in both animal and human studies. We found the susceptibility of cornu ammonis 1 and cornu ammonis 3 in ipsilesional hippocampus significantly increased over time, indicating age-related iron accumulation in these regions. The two regions in the contralesional hemisphere also showed similar long-term trajectories after 24 weeks but without significant effect. Consistent with our results, previous MRI studies have reported a positive correlation between iron content and age in the hippocampus of humans^81,82^ and gerbils.^83^ The susceptibility of dorsal fornix in the ipsilesional and contralesional hemisphere and anterior commissure posterior part in contralesional hemisphere significantly decreased over time and the susceptibility loss peaks at 36 weeks followed by a recovering trend between week 36 to week 48. This potentially reveals age-related myelination or calcium accumulation before 36 weeks and demyelination or axon loss after 36 weeks. Interestingly, the susceptibility of the anterior commissure posterior part in the ipsilesional hemisphere also showed a similar long-term trajectory after 24 weeks. Cellular components in white matter, including macroglial cells and axons, have receptors and channels on the cell membrane that allow calcium to pass. The calcium overload in white matter was reported in stroke mice and the calcium dyshomeostasis of white matter is age-dependent.^84,85^ Our results may provide new evidence for age-related calcium dyshomeostasis in healthy animals. Sturrock *et al*.^86^ reported a significant myelin increase in the posterior part of Anterior Commissure in 18-month-old mice compared with 5-month-old mice. Their group also demonstrated axonal abnormalities including axon degeneration and myelin vacuolation in mice aged 12 months and over^87^ and in aged (25–35 years) rhesus monkeys.^88^ A significant loss of myelinated nerve fibres in the fornix was found in 33-year-old rhesus monkeys.^89^ The dorsal fornix is a principal efferent pathway of the hippocampus, originating in the hippocampus and ending at the anterior commissure.^90^ Our result suggests age-related changes in the hippocampus—dorsal fornix—anterior commissure pathway, an essential pathway for memory encoding and integration.

Our findings suggest that both age-related iron accumulation in the hippocampus and calcium overload in dorsal fornix and anterior commissure are associated with demyelination in dorsal fornix and anterior commissure. Structural changes in these regions are strongly linked to cognitive impairment and memory decline.^91-93^ Calcium overload in white matter can harm glia and axons due to abnormal activity of calcium channels opened by ATP and glutamate, leading to white matter damage.^85^ White matter is vulnerable to iron due to the lack of antioxidants,^94,95^ and the oxidative stress from excess iron can induce macrophages to damage the myelin sheath of white matter.^96^ Excess iron can induce mitochondrial dysfunction, leading to the degeneration of axons.^97-99^ It was reported that age-related iron accumulation can trigger the disruption of nearby white matter in the human brain.^100^ Accumulation of iron in the hippocampus^81,101,102^ and the white matter change in the fornix^91,103,104^ and anterior commissure^88^ are associated with memory decline and cognitive impairment.

Primary somatosensory cortex dysgranular zone, and posterorostral part and posterocaudal part of parietal cortex in contralesional hemisphere showed significant susceptibility increase over time, suggesting iron accumulation or demyelination in subfields of the parietal cortex. Previous findings have shown older adults have a less extensive functional network in the parietal cortex during memory retrieval tasks.^105^ Our results are consistent with these findings, given negative correlations between functional connectivity and iron levels,^106-108^ and white matter connectivity support of functional connectivity.^109-111^

The granule cell level of the cerebellum showed an age-related susceptibility increase in the contralesional hemisphere, indicating iron accumulation in the regions. In agreement with our result, previous studies have reported age-related iron accumulation in the cerebellum of mice^112^ and humans.^113,114^ Beyond these regions, age-related susceptibility decreases in the ectorhinal cortex, interpeduncular nucleus and glomerular layer of the accessory olfactory bulb, and susceptibility increases in perirhinal area 36 (increase) were found. No age-related iron level or white matter changes have been previously reported in these areas so further exploration is warranted.

There are several limitations to this study. First, QSM provides a non-specific measure of iron, calcium and white matter, and can also be influenced by tissue geometry and other bio-metals. An important future direction is to apply susceptibility source separation approaches to better disentangle paramagnetic and diamagnetic contributions to bulk susceptibility, which may improve biological interpretability and help clarify which sources drive the observed longitudinal changes.^115-117^ Second, the small sample size limits statistical power, such that only regions with large effects reached significance. Third, we normalized QSM values to the cohort mean, which facilitates comparisons across ROIs but may obscure subtle group-specific differences; alternative strategies (e.g. normalization to controls) could be explored in future work.^118,119^ Finally, our analyses focused on the first two leading TCAM factors, which capture the most stable variance patterns but may miss subtle effects in lower-variance components, which are more likely to reflect noise or idiosyncratic variability.

## Conclusion

In this study, we investigated spatiotemporal volume and QSM changes in a rat model of stroke using TCAM, a tensor-based dimensionality reduction technique. We observed trajectories of QSM changes shared between the stroke and sham groups, potentially revealing age-related brain iron and calcium deposition and demyelination. Further histological assays are warranted to understand the mechanisms underlying these long-term structural changes in the brain.

## Supplementary Material

fcag182_Supplementary_Data

## Data Availability

The analysis code supporting the findings of this study is available as Jupyter notebooks in the Supplementary material. The datasets supporting the findings of this study are available from the corresponding author upon reasonable request.
